# Supplementary material for: An orthogonal TRAP enables intersectional genetic access to activated neurons in the mouse brain
Source: bioRxiv. 2026 Jan 6:2025.11.30.691330. Preprint. [Version 2] doi: 10.64898/2025.11.30.691330 (PMC12803082; doi:10.64898/2025.11.30.691330)
Supplement: Supplement 1 [file NIHPP2025.11.30.691330v2-supplement-1.pdf]

bioRxiv preprint doi: https://doi.org/10.64898/2025.11.30.691330; this version posted January 6, 2026. The copyright holder for this preprint (which was not certified by peer review) is the author/funder, who has granted bioRxiv a license to display the preprint in perpetuity. It is made available under aCC-BY 4.0 International license.

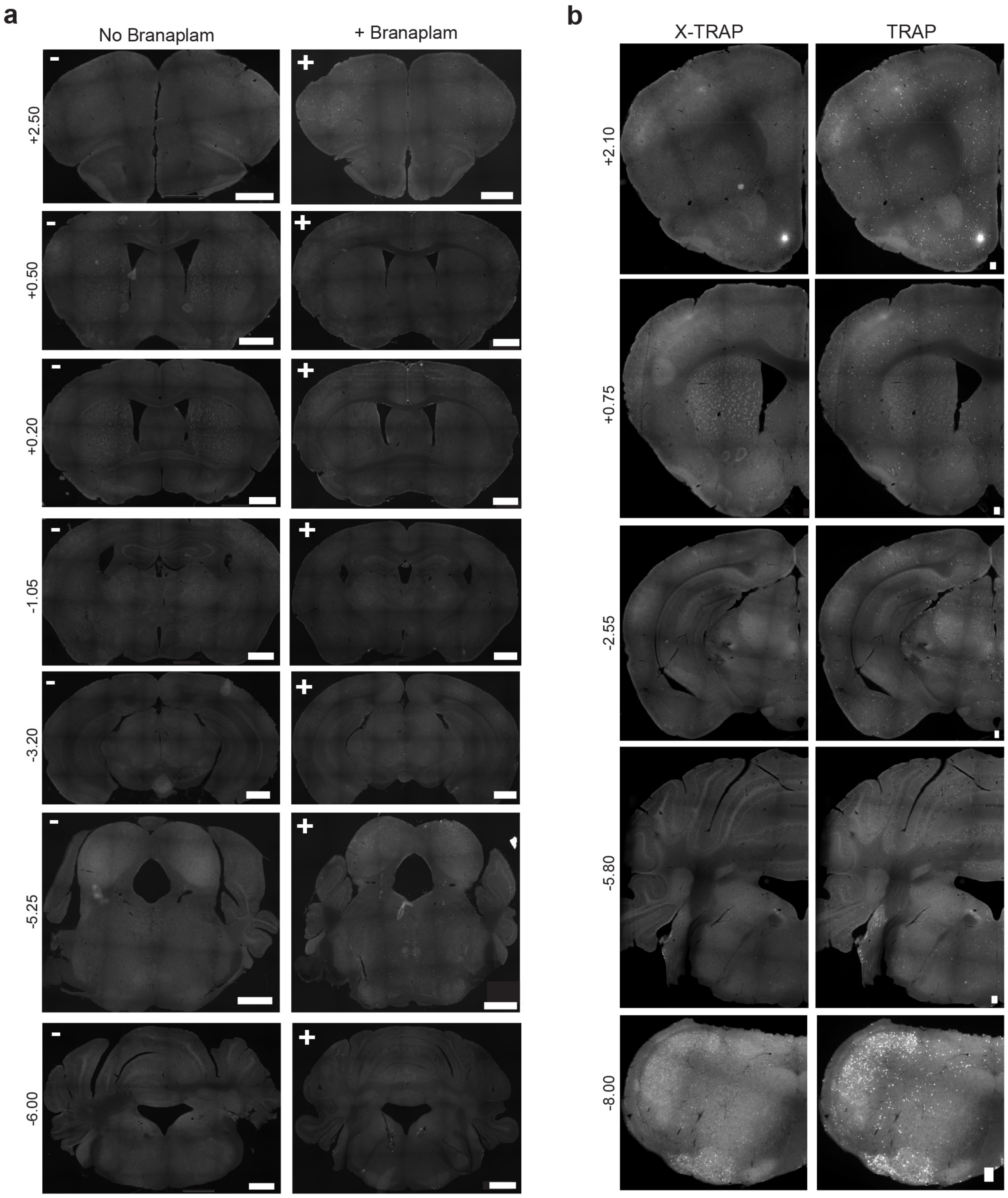

Supplementary Figure 1. Homecage recombination by X-TRAP and TRAP. a. Homecage recombination without (left) and with (right) branaplam treatment. Shown is Tomato fluorescence in an X-TRAP mouse crossed to a Flp-Tomato reporter. Note there are no cells without branaplam, and only sparse recombination after branaplam.The numbers at left are the distance from bregma of the slice. Scale bars are 1 mm. **b.** Homecage recombination with X-TRAP (left) versus TRAP (right) in the same mouse. Note that there is generally less recombination with X-TRAP. Scale bars are 0.1 mm.

bioRxiv preprint doi: https://doi.org/10.64898/2025.11.30.691330; this version posted January 6, 2026. The copyright holder for this preprint (which was not certified by peer review) is the author/funder, who has granted bioRxiv a license to display the preprint in perpetuity. It is made available under aCC-BY 4.0 International license.

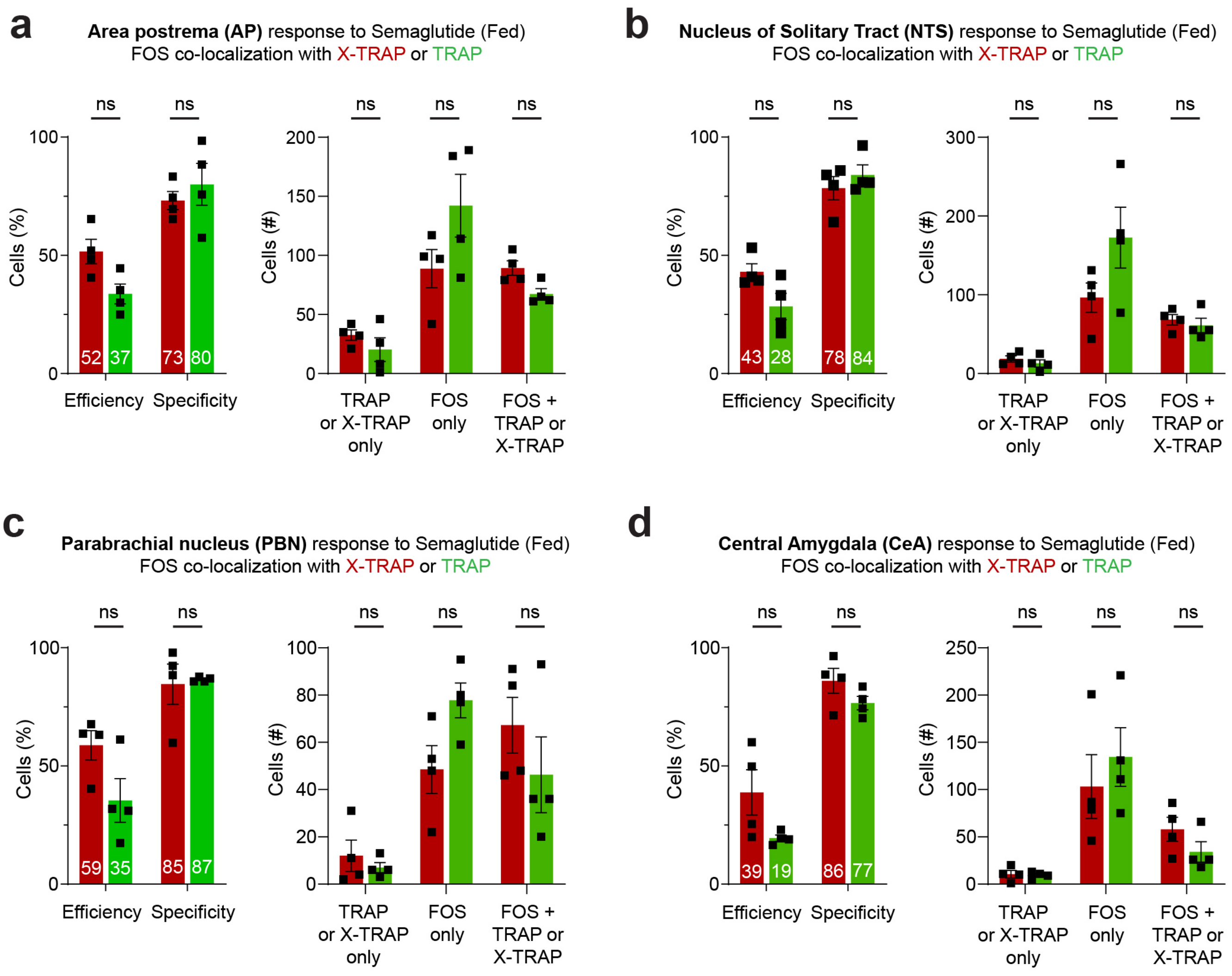

Supplementary Figure 2. X-TRAP and TRAP label semaglutide-activated neurons with similar specificity and efficiency in fed mice. a-d. Left. Shown in each panel is the efficiency (i.e. % FOS+ cells that are also labelled by X-TRAP (red) or TRAP (green)) and specificity (i.e. % X-TRAP (red) or TRAP (green) cells that are also labelled by FOS). **Right.** Shown in each panel is the number of cells with each labelling. Note that, in this experiment, the X-TRAP and TRAP labelling were performed in different mice. Thus, for example, the red bar in the “FOS only” column is the number of FOS+ cells that were not labelled by X-TRAP.

bioRxiv preprint doi: https://doi.org/10.64898/2025.11.30.691330; this version posted January 6, 2026. The copyright holder for this preprint (which was not certified by peer review) is the author/funder, who has granted bioRxiv a license to display the preprint in perpetuity. It is made available under aCC-BY 4.0 International license.

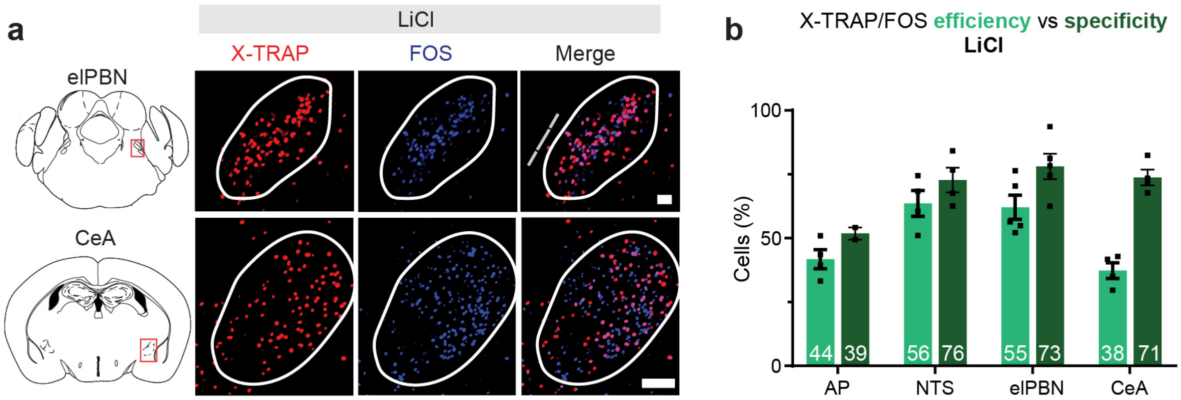

Supplementary Figure 3. Labeling of LiCl activated neurons by X-TRAP. a. Co-localization between X-TRAP recombination and endogenous FOS induced by LiCl treatment. **b.** Efficiency (percentage of FOS+ cells that are Tomato+) and specificity (percentage of Tomato+ cells that are FOS+) for LiCl-induced labelling. Scale bars are 0.1 mm. Data are mean ± sem.

bioRxiv preprint doi: https://doi.org/10.64898/2025.11.30.691330; this version posted January 6, 2026. The copyright holder for this preprint (which was not certified by peer review) is the author/funder, who has granted bioRxiv a license to display the preprint in perpetuity. It is made available under aCC-BY 4.0 International license.

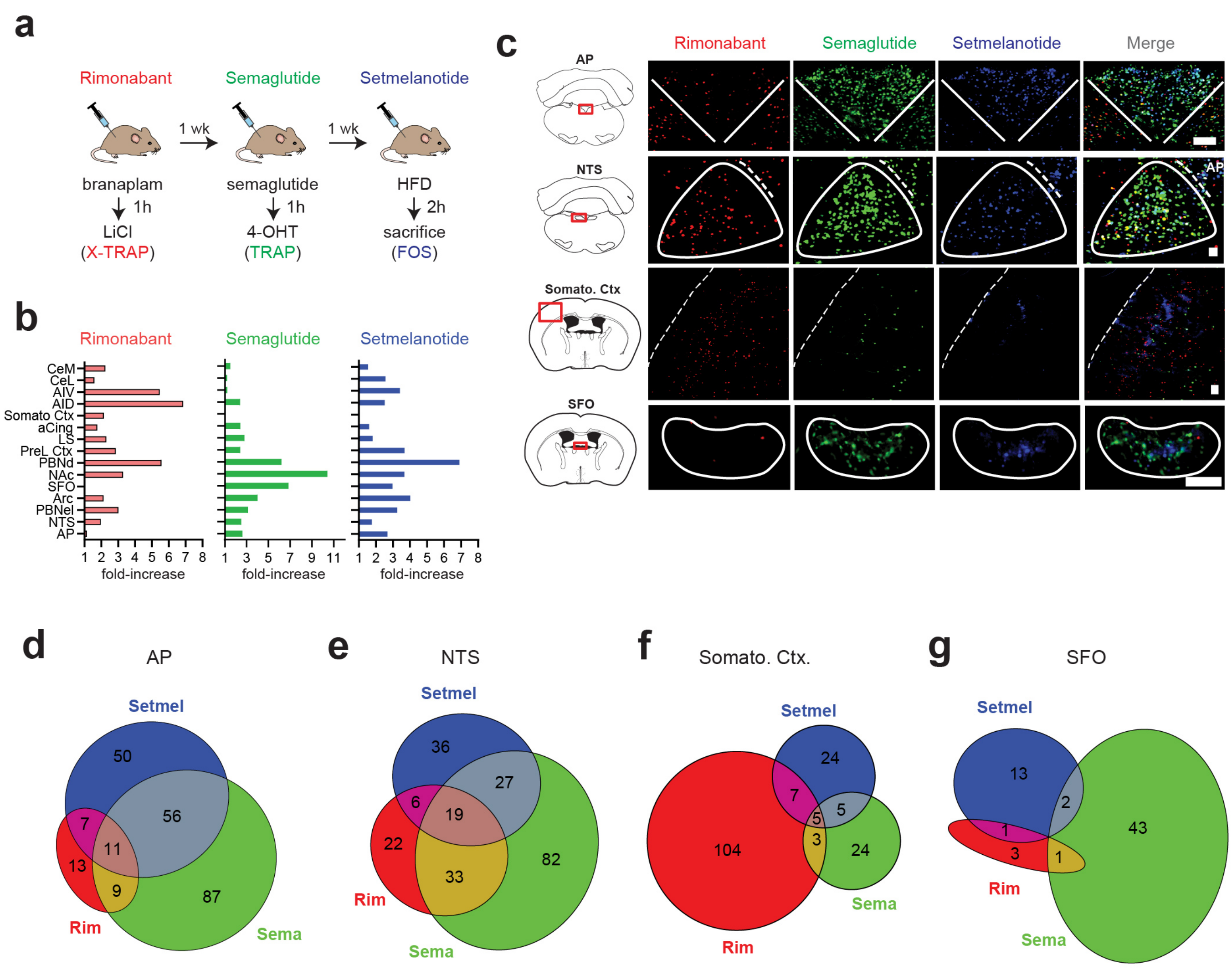

Supplementary Figure 4: Mapping brainwide responses to weight-reducing drugs. a. Protocol for sequential labelling of neurons activated by three drugs. **b.** Number of cells labelled across different brain regions, expressed as the fold-increase relative to vehicle injected controls. Abbreviations: PVH - Paraventricular Hypothalamus, BNST - Bed Nucleus of the Stria Terminalis, CeM - Central Amygdala, medial part, CeL - Central Amygdala, lateral part, AIV - Anterior Insula, ventral part, AID - Anterior Insula, dorsal part, Som. Ctx - Somatosensory Cortex, aCing - Anterior Cingulate Cortex, LS - Lateral Septum, PreL. Ctx - Prelimbic Cortex, dPBN - Lateral Parabranchial Nucleus, dorsal part, NAc - Nucleus Accumbens, SFO - Subfornical Organ, Arc - Arcuate Hypothalamus, elPBN - Lateral Parabranchial Nucleus, external lateral part, NTS - Nucleus of the Tractus Solitarius, AP - Area Postrema. **c.** Examples of labelling in selected brain regions in response to drug injection. **d-g.** The single-cell overlap between the neurons activated by these drugs in the brain regions indicated. Scale bars are 0.1 mm.
